# Ten simple rules for researchers who want to develop web apps

**DOI:** 10.1371/journal.pcbi.1009663

**Published:** 2022-01-06

**Authors:** Sheila M. Saia, Natalie G. Nelson, Sierra N. Young, Stanton Parham, Micah Vandegrift

**Affiliations:** 1 State Climate Office of North Carolina, Raleigh, North Carolina, United States of America; 2 Department of Biological and Agricultural Engineering, North Carolina State University, Raleigh, North Carolina, United States of America; 3 Center for Geospatial Analytics, North Carolina State University, Raleigh, North Carolina, United States of America; 4 North Carolina State University Libraries, North Carolina State University, Raleigh, North Carolina, United States of America; Dassault Systemes BIOVIA, UNITED STATES

## Introduction

Web applications, also known as web apps, are increasingly common in the research communication portfolios of those working in the life sciences (e.g., [1]) and physical sciences (e.g., [2–4]). Web apps help disseminate research findings and present research outputs in ways that are accessible and meaningful to the general public—from individuals, to governments, to companies. Specifically, web apps enable exploration of scenario testing and policy analysis (i.e., to answer “what if?”) as well as coevolution of scientific and public knowledge [5,6]. However, the majority of researchers developing web apps receive little formal training or technical guidance on how to develop and evaluate the effectiveness of their web-based decision support tools. Take some of us for example. We (Saia and Nelson) are agricultural and environmental engineers with little experience in web app development, but we are interested in creating web apps to support sustainable aquaculture production in the Southeast. We had user (i.e., shellfish growers) interest, a goal in mind (i.e., develop a new forecast product and decision support tool for shellfish aquaculturalists), and received funding to support this work. Yet, we experienced several unexpected hurdles from the start of our project that ended up being fairly common hiccups to the seasoned web app developers among us (Parham). As a result, we share the following 10 simple rules, which highlight take-home messages, including lessons learned and practical tips, of our experience as burgeoning web app developers. We hope researchers interested in developing web apps draw insights from our (in)experience as they set out on their decision support tool development journey.

**Fig 1:**
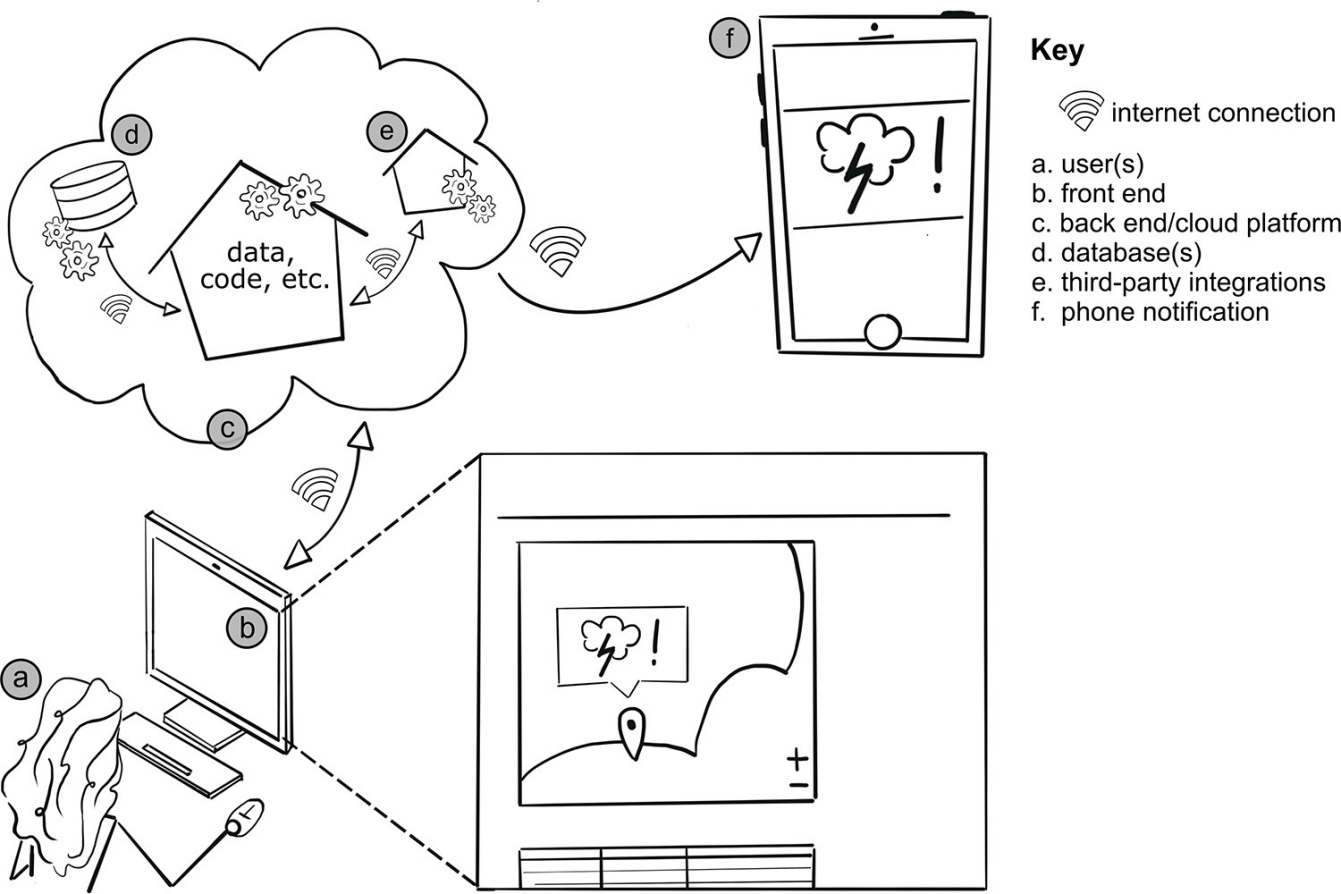
Major components of a web application (web app). In this line drawing, the (a) user is shown interacting with the (b) front end of the web app. The front end is updated based on changes that occur in the back end (c and d). Updates that occur in the back end can also trigger other actions, such as (f) text or email notifications via (e) third-party integrations. *Image credit*: *Sheila M*. *Saia (CC BY 4*.*0)*.

We focus on web apps, rather than mobile phone applications, because advances in web app coding frameworks make it possible to seamlessly scale web apps across multiple devices (e.g., phones, computers, and tablets). Web apps provide interactive services that can be accessed by web browsers [7]. Here, we further define web apps as dynamic tools that allow users to perform a task, although we acknowledge that others may define web apps differently. Web developers often separate web apps into two main components: the front end and the back end (Fig 1). There are some exceptions to this design. For example, some web apps are front end only and require no dedicated back end (e.g., single-page applications like https://github.com/igvteam/igv.js) [8]. These can usually be hosted on free third-party services such as GitHub Pages. The front end represents everything the user sees on their device screen (Fig 1B, 1F), while the back end represents parts of the web app that only the web developers see (Fig 1C, 1D). The back end typically includes (1) scripts (i.e., computer code) written in a back end language (e.g., Java and Python) to support the front end appearance and back end functionality of the web app (i.e., how periodic updates are made to the front end); (2) databases (e.g., MySQL) to store data for the web app and its users; and (3) web services (e.g., Google Cloud Platform, https://cloud.google.com) to present the updated web app to users and connect the user’s front end experience with the back end tasks via the web app (Fig 1C–1E). Most commonly, a trained or experienced web developer will specialize in one particular component; however, some web developers may specialize in the full stack, which refers to the front end and back end of the web app combined.

Web apps are powerful tools for increasing the accessibility and approachability of research data and findings because they leverage recent advancements in interactive web browsers to support browser-based user interfaces without the need for download and installation. Examples of web apps range from interactive maps depicting disease transmission (e.g., [9]), marine health (e.g., [10,11]), natural hazards (e.g., [12,13]), and pest infestations (e.g., [14,15]) to bioinformatics resource collections (e.g., [16]), to omics data analysis platforms (e.g., [17]), and to citation visualization tools (e.g., [18]), among others. Throughout this ten simple rules paper, we focus on web apps that help stakeholders make decisions by improving their access to information. However, web apps can be beneficial beyond their use by stakeholders. As an example, web apps can support research by improving the sharing of results and facilitating communications between scientists (e.g., [12,13,19]). In this ten simple rules paper, we reflect on our experiences developing the aforementioned decision support tool and web app, called “ShellCast” (https://go.ncsu.edu/shellcast), as well as how our experience applies more broadly to researchers venturing into web development. ShellCast is a noncommercial, open-source product (i.e., ShellCast source code is freely accessible and editable by anyone), but researchers interested in commercializing their web apps can look to other articles in the ten simple rules collection [20,21].

We were motivated to develop ShellCast after speaking with shellfish growers, state aquaculture management program staff, regional weather forecast staff, and university Extension agents, who all told us that there were no specialized weather apps to (1) help shellfish growers interpret daily rainfall forecasts; and (2) prepare their operations for temporary shellfish harvest area closures. These temporary closures, which prohibit shellfish harvesting in a particular area for approximately 1 to 2 weeks, are issued by the state of North Carolina to protect consumers from ingesting contaminated seafood after large rainfall events that flush harmful bacteria into estuarine waters [22–24]. Briefly, ShellCast users can sign up to create an account and receive a text message and/or email notification (Fig 1F) at the start of each day that will alert them of imminent rainfall events over the next 1 to 3 days, the occurrence of which can result in restrictions to their shellfish harvesting operations. By creating an account with the web app, users select a geographic location or locations that they would like to receive notifications for and their preferred notification type (i.e., text message, email, or both). Users can also view their notifications and notification locations on the web app main page (Fig 1B). There are many back end aspects to ShellCast that users do not see (Fig 1C–1E). These include but are not limited to: (1) timed running of web app back end tasks and code, also known as cron jobs, which update the web app database(s) each day at 7 AM; and (2) timed interactions between the web app database(s) and third-party notification providers (i.e., email and text message notification services).

We used Google Cloud Platform to develop ShellCast because of certain requirements (e.g., text and email message notifications) that would have been more difficult and/or costly, but not impossible, to implement with other web app frameworks. Alternative frameworks include Shiny (https://www.shinyapps.io), Dash (https://plotly.com/dash), ESRI StoryMaps (https://storymaps.arcgis.com), Tableau (https://www.tableau.com), HiCharts (https://www.highcharts.com), and PowerBI (https://powerbi.microsoft.com). As an example, web app email authentication is possible with Shiny, an open-source framework, but this service is only provided under higher web app hosting pricing plans. However, we encourage novice web developers to consider their project goals and check out these user-friendly platforms for developing interactive, engaging, and research-driven web apps.

Furthermore, in our case, there was a clear need for a web app that reduced the uncertainty of managing shellfish growing operations in coastal North Carolina. Prior to web app development, we recommend researchers ask: Is there demand for the proposed web app? Does a similar web app already exist that you could contribute to instead? Do we have the resources to maintain the app for years to come? Thinking about the utility and sustainability of the web app in the long term needs to be considered from the start and is key to developing an impact web app.

### Rule 1: Start with user-centered design

**Fig 2:**
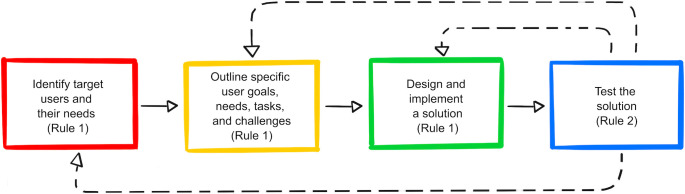
The four phases of UCD with associated rules. Dashed lines represent feedback in the iterative process. UCD, user-centered design*. Figure adapted by Sierra N. Young and Sheila M. Saia from [27].*

An idea for a web app, no matter how useful and wonderful it may seem, will not be of much use if you cannot articulate who is going to use your web app and what they will do with it. In the case of a decision support tool, it is especially important to know how your users will go about making decisions using your web app [25]. This process of designing a web app around what the user wants is broadly known as user-centered design (UCD). UCD is an iterative approach to design that focuses on understanding the user at all stages of the design and development phase [26,27]. The specific methods and processes implemented with UCD may vary by the project type and application, but, in general, UCD encompasses four iterative phases (see Fig 2): (1) understanding the context of use; (2) specifying user requirements; (3) designing solutions; and (4) evaluating the solution against user requirements [28].

The first two phases of UCD require conducting research that will help you understand the target users and tasks for your app, emphasizing that it is critical to consider users and their tasks early on in the design process, before jumping directly into web app development. The first phase focuses on understanding who, why, and under which circumstances users will actually use your web app, while the second phase focuses on the specific user goals, needs, tasks, and challenges that will shape the requirements of the web app. There are multiple methods that can be implemented during these phases, such as contextual inquiries, user interviews, task modeling, developing personas, and user and task analysis [29–31]. Many of these methods require interaction with potential users of your app. As an example, task-centered design, which is widely used for web app development [31,32], can be implemented to identify which specific steps must be taken by the user to meet their needs. If you have trouble finding potential users, this is a good indication that you may need to rethink your web app concept. Additionally, these research-focused phases offer an opportunity to determine whether or not a web app is the most appropriate method for implementing your proposed solution.

The third phase of UCD focuses on designing and implementing solutions. Depending on how far along in the design process your team is, this solution may take the form of a simple mock-up or wireframe or a fully functioning app prototype. It is unlikely you will get the web app design right the first time; therefore, getting feedback on low-stakes mock-ups early in the design process will save you time, money, and resources (see Rule 2). Admittedly, we (Saia and Nelson) did not include mock-ups in the first version of the ShellCast contract draft (S1 Text; see Rule 5) and were later advised by (very patient) Office of Information Technologies staff at our institution that it was imperative for users and web developers to see mock-ups prior to the creation of the web app. To illustrate how contracts and mock-ups are drafted in practice, we offer the final version of the ShellCast contract complete with mock-ups (S2 Text, S1 Fig). Finally, in the fourth phase of UCD, all designs developed during the development process must be tested and evaluated against user requirements (see user testing and evaluation in Rule 2).

### Rule 2: Test early, test often

Continuous testing is critical throughout the development process because it ensures that your web app is working as intended and is usable. Web apps must be checked for a variety of factors, including functionality, compatibility, security, and usability. During the software development phase, researchers should conduct functionality tests to ensure that software requirements are met. Functionality tests include evaluating individual units (e.g., forms, links, client page, and server page), unit integration (e.g., redirects and client/server couplings) as well as full system or end-to-end functionality, the latter testing all layers of an application in a single workflow [33]. These types of tests can often be automated with tools such as Cypress (www.cypress.io) or Selenium (www.selenium.dev) and are critical for finding bugs or other software-related issues. Automated functionality testing cannot always fully replicate the user experience, so be sure to supplement this type of software functionality testing with evaluation and testing by real users.

User testing and evaluation should be conducted throughout the web app development process and include a diverse range of potential users. User testing is important because it allows the web development team (including researchers, like us) to iterate on the web app design and ensure that it meets user expectations. There are commercially available services and companies dedicated to conducting comprehensive user tests; however, these services have the potential to slow down development. If budget or time constraints prevent you from using these services, you can conduct your own user testing with a little guidance (e.g., [31]). In general, there are three main types of user testing: (1) formative evaluations, which are performed during iterative design to find web app usability issues to be fixed during the next iteration; (2) field studies, which find problems in web app use contexts and collect qualitative observations; and (3) controlled experiments, which test hypotheses and collect quantitative observations about web app use [31,34]. In general, user testing requires finding actual users, selecting tasks for evaluation, providing users with a prototype web app for use, deciding what data to collect, choosing an evaluation method, and collecting data. There are many types of evaluation methods, each with their own purpose, pros, and cons. Common methods include surveys (chapter 5 of [34]), case studies (chapter 7 of [34]), focus groups (chapter 8 of [34]), interaction evaluation and measurement tools such as UXtweak (uxtweak.com) (see also chapter 23 of [35]), and online testing apps and services, such as User Testing (www.usertesting.com) or UsabilityHub (usabilityhub.com). A comprehensive survey of evaluation methods is outside the scope of this paper, but the resources provided in this section should provide enough guidance to get started on your user testing journey. In addition, if you find yourself evaluating a web application that provides new functionality, findings from formal user testing studies [34] may be publishable in an appropriate journal if the necessary steps are taken to design the experiments and protect participant privacy (see Rule 4).

We (Saia and Nelson) knew very little about user testing when developing ShellCast. Despite our limited knowledge, we understood that feedback was important and implemented two user testing periods using surveys administered via Google Forms. The goal of these surveys was to learn about potential issues that users might encounter when interacting with an initial version (user test #1, S3 Text) and improved version (user test #2, S4 Text) of ShellCast. While participants in these tests were not actual users of the web application (i.e., shellfish growers), they were colleagues in our field who have connections with actual users. The 2 ShellCast user testing surveys that we distributed early in the web app development process proved helpful in uncovering issues associated with signing up for an account, getting text notifications, and deciding which and how much information to convey to web app users. We are in the process of rolling out ShellCast to shellfish growers and conducting additional user testing through surveys and focus group discussions led by a professional facilitator.

### Rule 3: Make it accessible

To ensure that web apps can be used by all, it is important that researchers adhere to accessibility guidelines. Here, we consider accessibility not as a measure of openness as described by Findable, Accessible, Interoperable, and Reusable (FAIR) [36,37] research output guidelines, but rather as a measure of a web app’s utility to people of diverse abilities. Website accessibility is important because it helps ensure that a broad group of people will be able to use your app and also because there are laws mandating that your app and websites are accessible. Being based in the United States (US), we focus on US laws and standards in this ten simple rules paper. US-specific accessibility laws that impact researchers developing web apps include Title 2 [38] and Title 3 [39] of the Americans with Disabilities Act, associated web accessibility standards such as Section 504 through the US Department of Education Office of Civil Rights [40], and Revised Section 508 and 255 Guidelines of the Rehabilitation Act [41]. For researchers in the US, these legal standards are enforced by the US Department of Education’s Office of Civil Rights. These standards incorporate Web Content Accessibility Guidelines [42] developed by the international World Wide Web Consortium Web Accessibility Initiative; therefore, researchers outside of the US can look to these web accessibility guidelines too. If these laws and standards are not met, accessibility conformance can be enforced by informal complaints made directly to the web developer or formal complaints made through the Office of Civil Rights or through lawsuit to the university.

You should plan for accessibility as early as possible in the web app development process. This includes taking time early on to ensure that your web app is designed so it can be accessed by assistive technologies such as dictation software, screen readers, refreshable braille displays, and many others. For example, information provided in a map can also be made available in a table format (Fig 1B zoomed inset), the latter of which is more accessible to screen readers. You can also practice accessibility when developing surveys and feedback forms [43], captioning web app–related videos, and including alt text along with all images. Alt text is text that describes an image (nontextual) and is assigned as an image attribute in the front end HTML tag for the image (e.g., <img src = "picture.png" alt = "A picture">; in the web app back end language [44]). Assistive technologies like screen readers rely on the image attribute to communicate meaning to their users. Some of the most common web accessibility issues (e.g., low color contrast, unlabeled form fields, and no alt text or video captions) are fairly easy for web developers to fix [45].

Digital accessibility standards are fairly new and can be confusing, especially if you have little or no experience navigating them. Second to planning ahead, researchers can refer to Web Content Accessibility Guidelines [42]. Third, researchers can ask web accessibility coordinators for help reviewing and addressing potential web app accessibility issues (see Rule 7). The organizational structure of web accessibility coordinators at each institution is unique; however, these staff are often based in a researcher’s office of information technology, office of diversity and inclusion, office of disability resources, office of communications, or office of digital accessibility. Last but not least, web app developers can use web accessibility evaluation tools to scan their web app for accessibility issues and implement solutions to these issues via updates to front end design and back end scripts. Two example web accessibility evaluation tools include the pope.tech platform (https://pope.th) and the ANDI bookmarklet (https://github.com/SSAgov/ANDI). Programs like Color Oracle (https://colororacle.org) can help you check that web app graphics are color blind friendly. During the development of ShellCast, we scanned the application with pope.tech and discovered the contrast of our colors needed to be increased, which we likely would have never realized had we not used the pope.tech tool.

### Rule 4: Protect your users

Researchers developing web apps have a responsibility to meet modern web standards for user security, which include (1) protecting information that users share; and (2) being transparent about how data collected through the web app will be used. If based in the European Union, you must adhere to strict data privacy laws laid out in the EU’s General Data Protection Regulation (GDPR [46,47]). However, we recommend non-European–based researchers (like us) do their best to meet GDPR requirements because they protect the user and ensure that the web app is globally inclusive. Depending on the scope of your web app, researchers in the US may look to notable privacy protection laws including Health Insurance Portability and Accountability Act, Family Educational Rights and Privacy Act, Children Online Privacy Protection Act, and California Consumer Privacy Act. Web app security and privacy is especially important if users sign up, log in, and receive a service because information collected during this process may include personal identifiers like email addresses, phone numbers, mailing addresses, and other personal information. In our case, users can log into ShellCast, set up a profile, add map pins, and select text message and/or email notification preferences.

There are several ways researchers can put security and privacy protections into practice. First, you can leverage third-party integrations including sign up/sign in using Gmail, Facebook, Microsoft, Twitter, etc., because these services will manage passwords for you. You can also use cloud-based web services to offload typical security maintenance, thereby ensuring that your web app is deployed with the latest web security updates. In our case, our institution has access to Gmail and Google Cloud Platform, so ShellCast is built with these services. It is also important to have Transport Layer Security (TLS) to encrypt user inputs and keep them safe from hackers. From the user’s point of view, this looks like a “https://” web app hyperlink rather than the less secure “http://” hyperlink. Let’s Encrypt provides a basic level of TLS encryption at no cost that is appropriate for most needs. Second, you can include a privacy policy on your website that includes details on how information will be protected and used by the researchers. Privacy statement starter templates can be found online (e.g., [48]). As an example, you can view the ShellCast privacy policy (S5 Text). While it may take some planning ahead, you may also consider giving users the ability to delete their account and download their data; this is included in the GDPR discussed above. Third, you can create a data management plan, include details on what data will be made public and who will be responsible for data stewardship, and share that data management plan along with your web app documentation. Researchers can look to existing data management plan resources (DMPTool, https://dmptool.org; [49]) and web app management plan references mentioned in Rule 10. Fourth, if you expect to publish user feedback in peer-reviewed publications, you must get ethical approval, for example, from an institutional review board, before doing so. Last but not least, we endorse proactive, transparent, and ethical data management, as described by several others [50–53]. Ethical data management puts data privacy and data governance needs of the users and broader community first.

### Rule 5: Hire a web developer or become one

Researchers interested in creating web apps can contract out for web development. Doing so will improve web app functionality and professional appearance since the development firm will put together a team of specialists to work on your web app. Typically, this team will include a project manager, back end developer, front end developer, and possibly a graphic designer and documentation writer. If you are interested in contracting a web development firm, your first step will be to develop a request for proposals (RFPs) that will then be posted and advertised by your institution. Web development firms will then submit any follow up or clarifying questions, which you will need to answer so your institution can post your responses along with the public RFP. After reading your responses, web development firms will then submit proposals and budgets to your institution for your consideration. You will then choose which firm to contract with based on these proposals. Importantly, take time to think through web app tasks before writing and publishing the RFP. The RFP must be extremely precise and specific; it should outline all expectations for the web app, including its appearance and functionality (see Rule 1). If functions or features of the web app need to be adjusted at a later point, a contract renegotiation may be necessary. From our experience, if you are inexperienced in web app development, you may struggle to prepare an accurate and fully specified RFP, which can create a risky situation since you may go into contract for work that is not reflective of what you seek to accomplish. In addition to the challenges that come with preparing a precise RFP, budgets associated with web development firm projects can be large, as you are paying the salaries of a team of expert specialists. See the Supporting information for the early (S1 Text) and final (S2 Text) versions of our RFP; the final version includes edits that were made in response to questions from prospective web development firms.

Despite only having budgeted $20,000 USD for all web app–related expenses, we received proposed project budgets ranging from $60,000 USD to $180,000 USD. The more specificity you provide in the RFP, the smaller the proposed project budget ranges will be. Confronted with these outsized proposed budgets relative to our available funds, we explored alternatives. After going through this process, we learned that we could ask our institution to post the RFP on our local small business association email list, small business and technology development center email list (e.g., https://sbtdc.org/offices/ncsu), and on popular freelance job websites such as Fiverr (https://www.fiverr.com/) or Upwork (https://www.upwork.com/), among others.

Rather than having to work through the RFP process, we ideally would have identified a qualified web developer experienced in the type of web application we were interested in creating and then worked alongside the developer to outline expectations and needed features. From our experience, we came to appreciate the need for institutions and research sponsors to provide more resources that support expert software development. As the subdiscipline of research software engineering (RSE) grows and becomes more established [54–56], permanent RSE positions across multiple institutional levels (i.e., from general consultants at the university level to specialized positions embedded within research groups) will hopefully become commonplace. Had a research software engineer been available at our institution, we could have avoided the RFP process and hurdles associated with vetting outside developer groups.

The second alternative we identified was to hire a computer science student, which is the option we ultimately went with for ShellCast. Although still in training, many undergraduate and graduate computer science students have the skills needed to develop web apps—plus, they are eager to gain practical experience. We were able to hire the student (Parham) on an hourly basis, which provided flexibility as we ventured into new territory and identified additional features and functions during the development process that we had not originally considered (because we are novice web developers). Had we contracted with a web development firm, we likely would have been limited in our ability to incorporate these new ideas generated by the web app development process into ShellCast without contract renegotiation. By hiring a student, we also avoided many of the administrative tasks and overhead costs associated with hiring an external freelance web developer or web development firm. Most importantly, we found that student applicants to the ShellCast team were eager to try out new tools that would best serve the project (instead of using tools they felt most comfortable with) and work with us despite our lesser experience. However, hiring a student to develop your app can come with sustainability challenges (see Rule 10).

Of course, rather than hire a web developer, you can become one yourself! Platforms like Shiny and ESRI StoryMaps offer user-friendly templates and tutorials to help novice developers create web apps. In our case, because ShellCast users needed to create accounts and receive text/email notifications, we needed external web development support. However, for researchers with introductory programming experience, there are many tools available that allow for the creation of simple web apps to present results and interactive data visualizations. Of course, undertaking web app development on your own will require time and energy that you may expend at the expense of other important tasks. In the process of developing ShellCast, we needed to outsource key elements of web app development in the interest of time efficiency, especially given that we were operating within the constraints of grant deadlines and managing other responsibilities outside of ShellCast. If we had more flexibility, we would have been more willing to take on the challenge of creating ShellCast without outsourcing a web developer. Additionally, since training students is part of our institution’s mission, we valued hiring a student over an outside organization because the student would have an opportunity to gain skills and experience as a member of our team. Therefore, deciding whether to develop a web app on your own versus hiring an outside developer will depend on your own expertise, timeline, and project vision.

### Rule 6: Expect expenses

To the unseasoned web application creator, the costs associated with maintaining an application can be surprising (e.g., see our web app budget underestimation story in Rule 5). At a minimum, plan to budget for a web developer, web hosting fees, Secure Sockets Layer certificate for web app encryption, domain name costs, and cloud computing services. Setting aside a “rainy day” or “emergency” fund is also wise, as unexpected issues can arise that may derail the development or use of your app.

Web hosting refers to a suite of services needed to make a web page available to users. When a web page is constructed, it is stored or “hosted” on an internet server (Fig 1C). Users accessing a web app enter the web address (i.e., URL) in their web browser (Fig 1B), and the web browser connects to the internet service (e.g., Google Cloud Platform) hosting the web app. You can think of web hosting fees as rent paid for the space your web app occupies on an internet server. Similar to rent, web hosting fees are paid over periods (i.e., annually or monthly) and depend on whether the web app is static or dynamic, how much storage space you need (e.g., 10 GB), the number of people you expect to use the web app at the same time (i.e., network traffic), and in the case of dynamic web apps, the additional computing resources required. Many web hosting providers also offer domain name purchasing (e.g., GoDaddy, Dream Host, and Google Domains). Domain names can be purchased from a domain registrar (e.g., GoDaddy, Dream Host, and Google Domains) on a recurring basis. Notably, many institutions, such as universities, provide internal web hosting and domain name services at low rates, so be sure to check with experts at your institution before spending your hard-earned grant money.

To create a static web page, only web hosting and domain name purchasing is needed. However, some web apps may require cloud computing (Fig 1C), which refers to the on-demand storage and processing of data over the internet without the need for direct, active management of those services by the web app developer. Common cloud computing providers include Google Cloud Platform, Amazon Web Services, and Microsoft Azure Cloud. Many of these services provide cloud computing cost estimators (e.g., Google Cloud Platform Pricing Calculator, https://cloud.google.com/products/calculator) and let you set budget alerts for each project. Additionally, if you plan to send emails or text messages as one of the functions of your application, expect to pay for each and every message sent using third-party integrations for emails (e.g., Mailchimp, SendGrid, and Mailgun) and short message service (SMS) texts (e.g., Twilio and Nexmo). Although the rates per message can be very low, these costs grow quickly as you scale up your web app. Lastly, we recommend you consider including funds to support user testing (see Rule 2), such as for contracted services or reimbursement for the testers’ time.

### Rule 7: Leverage institutional expertise

When starting on your web development journey, look to professionals at your institution for feedback and support; these staff may share helpful resources and be great sounding boards throughout web app development while also bringing diverse perspectives and skill sets to your project. These professionals include information technology staff, library staff, computer scientists, user-experience/user-design staff, graphic designers, web accessibility staff (see Rule 3), research software engineers (see Rule 5), and many more. If your institution does not offer RSE support, you may also benefit from including students from computer science or other related fields on your team (see Rule 5). These students are often looking for hands-on experience as they work toward the completion of their degree.

We found several professionals in the information technologies office, library, and communications office that supported our work on ShellCast. Specifically, our university has designated outreach technologies staff within the information technology office; these staff regularly meet with researchers and give them feedback on resources, tools, and services that are available to support university-related web app development. Keep in mind that getting feedback from institutional staff is complementary to, but does not replace, involving users in the web app development process (see Rules 1 and 2). In a series of meetings with our outreach technologies staff, we were introduced to mock-ups (see Rule 1), university supported web app structures (see the Introduction, Fig 1C–1E), database structures, web accessibility standards (see Rule 3), user privacy protection (see Rule 4), skills needed by the web developer to bring our app to fruition, user testing (see Rule 2), and much more. In addition to getting feedback from outreach technologies staff, we also contacted library staff to review the ShellCast web app documentation (see Rule 10). This was especially helpful because the university has staff (Vandegrift) who specialize in documentation, licensing, and sustainability of open-source software. Our funders required ShellCast to be open source, although we intended to pursue open-source standards all along. Additionally, we leveraged the expertise of a graphic designer in the communications office to help us develop the ShellCast logo as well as an infographic. These graphics enhanced the appearance of ShellCast and helped us explain how ShellCast works to members of the general public. In the end, working with a computer science student and in-house graphic designer kept us well within our budget.

### Rule 8: Track your progress with existing collaboration tools

There are a number of existing resources and collaboration tools to help researchers and web developers keep track of their work, plan out project milestones, and assign tasks. The specific collaboration tools you choose to use when developing web apps may depend on many factors including whether (1) the tool easily interfaces with other available tools and resources; (2) the tool has all (or most of) the functionality needed to manage the web app project; (3) collaborators have previous experience and recommend using the tool; and (4) your team has the resources (e.g., financial and computing) to use the collaboration tool. To keep track of changes to web app code, use version control [57–59]. You can also use Kanban project management tools (e.g., [60]) such as those provided through platforms like GitHub projects (https://github.com/features/project-management), Trello (https://trello.com), Teamwork (https://www.teamwork.com), Jira (https://www.atlassian.com/software/jira), and many others. Project management tools can help the web app team chart project milestones, create and assign tasks, and keep track of emerging issues.

While building the ShellCast web app, we used both Git and GitHub to collaborate on and keep track of code. We created a GitHub project within the ShellCast web app repository and used this to track each team member’s progress on different tasks (also referred to as “issues” in the GitHub platform) as they moved from the “To Do” pile, to the “In Progress” pile, to the “Done” pile. We could comment on tasks in GitHub, which was helpful when referring back to past conversations and justifications for decisions even after tasks were completed. We could also use the issues to take notes and save helpful resources that we did not want to lose or could be important for new team members joining in the future. Importantly, keep in mind that when using an institution’s enterprise GitHub account, you will have to mirror your enterprise GitHub repository to a public GitHub repository should you wish to share your web app code openly. This is because the “public” setting on your enterprise GitHub repository is only public to folks within your institution. If we were to start over, we would have exclusively used a public GitHub repository.

Some of these same collaboration tools may also help you pursue open science. Reproducible and open work is often highly recommended by professional societies (e.g., [61]) and a requirement of federal funding; therefore, it’s important that you are aware of the expectations of your sponsors (e.g., [62]). Open and reproducible work may also be required by publishers (e.g., American Geophysical Union journals [63] and Public Library of Science (PLOS) journals [64]), which is important to consider early on should you wish to eventually publish a paper on your web app. The Carpentries offer several beginner-friendly, self-paced tutorials on version control, reproducible research, and programming languages widely used in open science at https://software-carpentry.org/lessons.

### Rule 9: Estimate task times, then double them (and then some)

We recommend generously estimating the time needed to develop a web application, especially if you are new to application development. Keep in mind that even small changes to a web app can lead to reconfigurations of database structures and back end web application logic, often resulting in seemingly minor changes requiring a considerable amount of time to complete. You should not be surprised if tasks will change or be carried out differently after user testing. Make sure you budget conservatively for time needed to revise the application after receiving feedback from user testing (see Rule 2) and to document the web app (see Rule 10). Furthermore, we acknowledge that estimating task times can be very difficult because they depend on a number of things including (1) the number of developers working on the web app; (2) the experience the developers have working with the technologies that your web app needs; (3) the size and complexity of the web app; (4) the specificity of the web app functionality (i.e., whether or not you know exactly what the web app will do, how it will look, and how it will behave); (5) project organization and efficiency and many other uncertainties that are tough to comprehensively list here. There are tools available to help with estimating time needed to complete a web development project (e.g., Konigi [65] and Astuteo Estimator [66]), although these tools still require the user to estimate time ranges for each task. Ivan and Despa offer useful estimates of task times, particularly related to maintaining web applications [67].

Our experiences were as follows. The initial development of ShellCast took our web developer (Parham) approximately 275 hours, with revisions following two rounds of user testing amounting to 75 hours. This time does not include time spent by our second web developer (Saia) to develop the ShellCast algorithm and get up to speed on connecting to and updating the ShellCast database. To provide some more context, ShellCast is a small web app with fairly simple functionality that had two initial developers. One developer (Parham) worked on the web app overall code infrastructure, database, notifications, hosting, and documentation, while the other developer (Saia) worked on developing the forecast calculations, database, and documentation. The majority of ShellCast development was completed by these two web developers, who each worked strictly on web development for approximately 20 hours per week during the 2020 summer semester (i.e., May 15 to August 15, 2020). Web development was Parham’s primary focus, while Saia worked on web development (20 hours per week), coordinated and administered user testing (5 hours per week), managed project milestones and tasks (2 hours per week), and carried out other non-ShellCast research duties (13 hours per week). In the 2020 fall semester (i.e., August 15 to December 15, 2020), both web developers each worked on ShellCast development for approximately 5 to 10 hours per week; Parham’s focus shifted back to coursework and Saia’s focus shifted back to other research duties. This timeline worked well for us because we clearly and specifically defined ShellCast requirements in the beginning of the project; however, we had to make some significant changes along the way after having more in-depth conversations with collaborators; these changes were separate from those we made based on user testing feedback.

To balance time and web app development needs, we checked in with one another weekly to discuss what tasks we were working on, the level of urgency of a particular task, if we had any issues that were preventing progress, and our plans for addressing these tasks in the coming week. To help keep track of these tasks, we created a shared document listing out all of our milestones. We also used GitHub projects and GitHub tags (see Rule 8) that we updated on an approximately monthly basis.

### Rule 10: Make it last: Plan for the long haul

The longevity of a web app depends on well-planned support (i.e., funding), maintenance, and documentation. Without proper planning, the impact of your web app will be cut short. In terms of support, web apps are commonly included in grants as a mechanism for disseminating research findings to stakeholders. In our limited US-based experience, proposals are rarely required to include plans on how a proposed web app will exist beyond the duration of the 1- to 5-year grant. Even when proposals include discussion of long-term web app support, the development phase often occurs toward the end of a project period, leaving little time for the web app to be discovered and used. By contrast, funders in the United Kingdom and EU often require a software management plan (e.g., [68,69]). To ensure long-term maintenance and utility of your web app, determine who will be designated as the web app maintainer(s) [58,70] and how long-term web app ownership and maintenance is defined [71].

Regarding documentation, we recommend budgeting time (and funds) for documenting your web app as well as incorporating documentation and project sustaining best practices (e.g., [72–74]). Whenever possible, build your web app using widely supported technologies and include a test suite (see Rule 2) to ensure that the web app code will function properly as an ensemble after you have made changes to the source code and web app dependencies (i.e., the software and code versions that your web app depends on to run). If your code relies on established R packages, Python libraries, or other software with particular version numbers, using a container system like Docker (e.g., [75]) or software environment like Conda (e.g., https://docs.conda.io/en/latest) is critical for helping to future-proof your code and support its replicability. Although fundamentally different, both container systems and software environments allow for preservation of version-dependent software libraries with your unique code. You can use platforms like Zenodo (https://about.zenodo.org) to permanently archive versions of your web app code and allow them to be cited via a digital object identifier (DOI).

For ShellCast, we explicitly included documentation of typical developer tasks in a DEVELOPER.md markdown file, included several other markdown files to document other important web app–related setup steps and tasks, and included a text file listing all the required R packages and Python libraries. We also documented ShellCast unit tests, and in the future, would like to implement automatic unit testing and deployment of the ShellCast web app using continuous integration platforms (e.g., Travis CI; https://travis-ci.org). While we did not use GitHub Actions (https://docs.github.com/en/actions) at the time we were developing ShellCast. This tool offers another helpful approach to automate tasks, including running your testing scripts. You can learn more about successful web app documentation, maintenance, and longevity as well as doing open and reproducible science from many resources available online (e.g., [76–82]), several of which are in the ten simple rules collection [21,37,57,70,72–74,83–87].

In the case of open-source web app development, researchers may wish to plan for and initiate involvement of the user community (see Rule 1), including researchers in related fields who are interested in maintaining the web app into the future. This group of interested users is often referred to as a maintainer community. Look to collaboration guidelines such as those proposed by The Mozilla Open Leaders Project for maintainer community best practices [88] or the Sustain online discussion board for open-source projects (https://discourse.sustainoss.org). Last but not least, we reiterate the importance of leveraging technology services offered through your university (e.g., domain names; see Rule 7) to avoid issues that could arise if your maintenance funds are limited (now or in the future). University technology services may impose some restrictions, like the lack of a public release option for a university-sponsored GitHub Enterprise account, but they at least provide a measure of expected sustainability and support from the organization’s IT and developer teams.

In many cases, app discoverability and longevity are linked and can be improved by using established cyberinfrastructure or building upon existing web apps. For example, Openscapes (https://openscapes.org) staff encourages researchers interested in open and reproducible science (including software and web app development) to ask themselves: “[Am] I being as open as I can be, am I being as inclusive as I can be, and will I be able to maintain what I’m starting?” [58,89]. Before beginning web app development, scan the web app landscape to see if there are similar open-source projects that could be adapted, rather than building a web app from scratch. Your subject specialist librarian is a great resource for starting this scan (see Rule 7) and can also help you navigate evolving practices in software citation (e.g., [90]), data publishing (e.g., [91]), and other emerging topics that are web app related. Most research libraries have subject specialists and/or functional experts (i.e., data management librarians) and can be generally supportive of many questions beyond providing resources for research. Since research infrastructure—the services, protocols, standards, and software that the academic ecosystem needs to perform its functions—is constantly modernizing and standardizing, these library staff can also help you improve the longevity of your web app. Specifically, they can share information and resources to build sustainable products (i.e., your web app) that are also interoperable across the landscape.

Since routine web app operation requires a domain name, web hosting, and cloud computing services, funds are needed for long-term support (see Rule 6). Researchers interested in developing web apps should acknowledge the need for continued support in proposals and outline potential funding sources that they can pursue to support web app longevity. Acknowledging that web apps require regular maintenance and enduring financial support demonstrates understanding of the realistic resources it takes for a web app to come to fruition, thus increasing the researcher’s credibility. Applying for alternate funding opportunities like Fund Open Source Software (https://fundoss.org), Chan Zuckerberg Initiative for Essential Open Source Software (https://chanzuckerberg.com/rfa/essential-open-source-software-for-science), Google Summer of Code (https://summerofcode.withgoogle.com), and Outreachy (https://www.outreachy.org), to name a few, can stretch the longevity of your web app. Of course, an alternative approach for sustaining a web app is to explore options for commercialization [20], which could cover the cost of web app expenses through advertisements or other user base-associated business models.

## Conclusions

Web apps serve as powerful tools to extend research findings to members of the public and research community, but their development is not easy. Successfully creating web apps for educational and outreach purposes requires teamwork with professionals that have diverse skill sets as well as careful and thoughtful planning to ensure that the web apps are relevant to end users, accessible to all, and long lasting. Here, we have outlined ten simple rules for researchers to consider as they venture out on their own web app development journeys, with several of these rules serving as “lessons learned” from our own personal experiences developing the web app, ShellCast. In summary, a good thought to keep in mind is build for usability, budget in flexibility, and begin maintenance plans from the start.

## Supporting information

S1 Fig ShellCast wireframes, including (a) main page map view when user is not signed in, (b) main page table view, (c) ShellCast “About” page, (d) user login page, (e) user notifications/profile page, and (f) main page map view when user is signed in (can see lease pin and click pin to see lease-specific information).
(PNG)

S1 Text First version of our RFPs contract “Scope of Work” section. RFP, request for proposals.
(DOCX)

S2 Text Final version of our RFPs contract “Scope of Work” section. RFP, request for proposals.
(DOCX)

S3 Text “Phase 0” ShellCast user testing survey questions. (DOCX)

S4 Text “Phase 1” ShellCast user testing survey questions. (DOCX)

S5 Text ShellCast privacy policy. (DOCX)

## References

[1] Bilimoria KY, Liu Y, Paruch JL, Zhou L, Kmiecik TE, Ko CY, et al. Development and Evaluation of the Universal ACS NSQIP Surgical Risk Calculator: A Decision Aid and Informed Consent Tool for Patients and Surgeons. J Am Coll Surg. 2013;217:833–42. doi: 10.1016/j.jamcollsurg.2013.07.385 24055383 PMC3805776

[2] Cooke T, Lingard H, Blismas N, Stranieri A. ToolSHeD TM: The development and evaluation of a decision support tool for health and safety in construction design. Eng Constr Archit Manag. 2008;15:336–51. doi: 10.1108/09699980810886847

[3] Horita FEA, Albuquerque JP de, Degrossi LC, Mendiondo EM, Ueyama J. Development of a spatial decision support system for flood risk management in Brazil that combines volunteered geographic information with wireless sensor networks. Comput Geosci 2015;80: 84–94. doi: 10.1016/j.cageo.2015.04.001

[4] O’Hara CC, Frazier M, Halpern BS. At-risk marine biodiversity faces extensive, expanding, and intensifying human impacts. Science. 2021;372:84–7. doi: 10.1126/science.abe6731 33795456

[5] Graffy EA, Booth NL. Linking environmental risk assessment and communication: an experiment in co-evolving scientific and social knowledge. International Journal of Global Environmental Issues. 2008;8:132–46. doi: 10.1504/IJGENVI.2008.017264

[6] Booth NL, Everman EJ, Kuo I-L, Sprague L, Murphy L. A Web-Based Decision Support System for Assessing Regional Water-Quality Conditions and Management Actions1: A Web-Based Decision Support System for Assessing Regional Water-Quality Conditions and Management Actions. J Am Water Resour Assoc. 2011;47:1136–50. doi: 10.1111/j.1752-1688.2011.00573.x 22457585 PMC3307623

[7] Shklar L, Rosen R. Web Application Architecture: Principles, Protocols, and Practices. Chichester, England: John Wiley and Sons, Inc; 2003.

[8] Robinson JT, Thorvaldsdóttir H, Winckler W, Guttman M, Lander E, Getz G, et al. Integrative genomics viewer. Nat Biotechnol. 2011;29:24–6. doi: 10.1038/nbt.1754 21221095 PMC3346182

[9] Center for Systems Science and Engineering at Johns Hopkins University. COVID-19 Map. In: Johns Hopkins Coronavirus Resource Center [Internet]. 2021 [cited 13 Aug 2021]. Available from: https://coronavirus.jhu.edu/map.html.

[10] Halpern BS, Longo C, Hardy D, McLeod KL, Samhouri JF, Katona SK, et al. An index to assess the health and benefits of the global ocean. Nature. 2012;488:615–20. doi: 10.1038/nature11397 22895186

[11] The Ocean Health Index. Historical Global Ocean Health Index Scores (2012–2020). 2020 [cited 13 Aug 2021]. Available from: https://ucsb.maps.arcgis.com/home/item.html?id=1f305abdc47a45bf867783c7419db6d0.

[12] Scheip CM, Wegmann KW. HazMapper: a global open-source natural hazard mapping application in Google Earth Engine. Nat Hazards Earth Syst Sci. 2021;21:1495–511. doi: 10.5194/nhess-21-1495-2021

[13] Incorporated Research Institutions for Seismology. IRIS Earthquake Browser 2021 [cited 13 Aug 2021]. Available from: http://ds.iris.edu/ieb/index.html?format=text&nodata=404&starttime=1970-01-01&endtime=2025-01-01&minmag=0&maxmag=10&mindepth=0&maxdepth=900&orderby=time-desc&src=usgs&limit=1000&maxlat=70.73&minlat=-70.73&maxlon=116.46&minlon=-116.46&zm=3&mt=ter.

[14] Keever T. Forecasting. In: Cucurbit Downy Mildew Forecasting Integrated Pest Management Pest Information Platform for Extension and Education (ipmPIPE) Project [Internet]. 2019 [cited 13 Aug 2021]. Available from: https://cdmpipe.mystagingwebsite.com/forecasting/.

[15] Cucurbit Downy Mildew Forecast. In: Cucurbit Downy Mildew Forecasting Integrated Pest Management Pest Information Platform for Extension and Education (ipmPIPE) Project [Internet]. 2019 [cited 13 Aug 2021]. Available from: https://cdm.ipmpipe.org/.

[16] Messina A, Fiannaca A, La Paglia L, La Rosa M, Urso A. BioGraph: a web application and a graph database for querying and analyzing bioinformatics resources. BMC Syst Biol. 2018;12:98. doi: 10.1186/s12918-018-0616-4 30458802 PMC6245492

[17] Arkin AP, Cottingham RW, Henry CS, Harris NL, Stevens RL, Maslov S, et al. KBase: The United States Department of Energy Systems Biology Knowledgebase. Nat Biotechnol. 2018;36:566–9. doi: 10.1038/nbt.4163 29979655 PMC6870991

[18] Tarnavsky-Eitan A, Smolyanksy E, Knaan-Harpaz I. Connected Papers. 2021 [cited 3 Aug 2021]. Available from: https://www.connectedpapers.com/.

[19] Tampa Bay Estuary Program. Piney Point Environmental Monitoring Dashboard. [cited 13 Aug 2021]. doi: 10.5281/zenodo.4666494

[20] Fletcher AC, Bourne PE. Ten Simple Rules To Commercialize Scientific Research. PLoS Comput Biol. 2012;8:e1002712. doi: 10.1371/journal.pcbi.1002712 23028299 PMC3459878

[21] Masum H, Rao A, Good BM, Todd MH, Edwards AM, Chan L, et al. Ten Simple Rules for Cultivating Open Science and Collaborative R&D. PLoS Comput Biol. 2013;9:e1003244. doi: 10.1371/journal.pcbi.1003244 24086123 PMC3784487

[22] Leight AK, Hood RR. Precipitation thresholds for fecal bacterial indicators in the Chesapeake Bay. Water Res. 2018;139:252–62. doi: 10.1016/j.watres.2018.04.004 29655096

[23] Leight AK, Hood R, Wood R, Brohawn K. Climate relationships to fecal bacterial densities in Maryland shellfish harvest waters. Water Res. 2016;89:270–81. doi: 10.1016/j.watres.2015.11.055 26689664

[24] Saia S, Nelson N, Young S, Hall S. Shellfish Leases and Harvest Closures Along the North Carolina Coast (AG-898). In: NC Cooperative Extension [Internet]. 2021 [cited 26 Aug 2021]. Available from: https://content.ces.ncsu.edu/shellfish-leases-and-harvest-closures-along-the-north-carolina-coast.

[25] Grêt-Regamey A, Sirén E, Brunner SH, Weibel B. Review of decision support tools to operationalize the ecosystem services concept. Ecosyst Serv. 2017;26:306–15. doi: 10.1016/j.ecoser.2016.10.012

[26] Abras C, Maloney-Krichmar D, Preece J. User-centered design. Encyclopedia of Human-Computer Interaction. Thousand Oaks: Sage Publications; 2021.

[27] Interaction Design Foundation. User Centered Design 2021 [cited 13 Aug 2021]. Available from: https://www.interaction-design.org/literature/topics/user-centered-design.

[28] User-Centered Design Basics. Department of Health and Human Services; 3 Apr 2017 [cited 13 Aug 2021]. Available from: https://www.usability.gov/what-and-why/user-centered-design.html.

[29] Pistoia Alliance. About the UX Methods. In: UXLS UX Toolkit for Life Sciences [Internet]. [cited 26 Aug 2021]. Available from: https://uxls.org/methods/.

[30] Benyon D. Designing interactive systems: A comprehensive guide to HCI, UX and interaction design. Pearson Education Limited (UK).

[31] Lewis C, Rieman J. Task-Centered User Interface Design: A Practical Introduction. Boulder, Colorado; 1993. Available from: http://hcibib.org/tcuid/tcuid.pdf.

[32] Ruiz J, Serral E, Snoeck M. Unifying Functional User Interface Design Principles. International Journal of Human–Computer Interaction. 2021;37: 47–67. doi: 10.1080/10447318.2020.1805876

[33] Di Lucca GA, Fasolino AR. Testing Web-based applications: The state of the art and future trends. Inf Softw Technol. 2006;48:1172–86. doi: 10.1016/j.infsof.2006.06.006

[34] Lazar J, Feng JH, Hochheiser H, editors. Research Methods in Human Computer Interaction. Morgan Kaufmann; 2010.

[35] Stone D, Jarrett C, Woodroffe M, Minocha S. User interface design and evaluation. Elsevier; 2005.

[36] Wilkinson MD, Dumontier M, IjJ A, Appleton G, Axton M, Baak A, et al. The FAIR Guiding Principles for scientific data management and stewardship. Scientific Data. 2016;3:160018. doi: 10.1038/sdata.2016.18 26978244 PMC4792175

[37] Garcia L, Batut B, Burke ML, Kuzak M, Psomopoulos F, Arcila R, et al. Ten simple rules for making training materials FAIR. Markel S, editor. PLoS Comput Biol. 2020;16:e1007854. doi: 10.1371/journal.pcbi.1007854 32437350 PMC7241697

[38] United States Department of Justice Civil Rights Division. State and Local Governments (Title II). In: Information and Technical Assistance on the Americans with Disabilities Act [Internet]. 2011 [cited 11 Mar 2021]. Available from: https://www.ada.gov/ada_title_II.htm.

[39] United States Department of Justice Civil Rights Division. Public Accommodations and Commercial Facilities (Title III). In: Information and Technical Assistance on the Americans with Disabilities Act [Internet]. 2011 [cited 11 Mar 2021]. Available from: https://www.ada.gov/ada_title_III.htm.

[40] United States Department of Education Office of Civil Rights. Regulation implementing Section 504 of the Rehabilitation Act of 1973, Title 34 Part 104. US Department of Education (ED); 1973 [cited 11 Mar 2021]. Available from: https://www2.ed.gov/policy/rights/reg/ocr/edlite-34cfr104.html.

[41] United States Access Board. About the ICT Accessibility 508 Standards and 255 Guidelines. In: US Access Board—Revised 508 Standards and 255 Guidelines [Internet]. 2017 [cited 11 Mar 2021]. Available from: https://www.access-board.gov/ict/.

[42] Web Accessibility Initiative (WAI). A customizable quick reference to Web Content Accessibility Guidelines (WCAG) 2 requirements (success criteria) and techniques. In: How to Meet WCAG (Quickref Reference) [Internet]. 2019 [cited 11 Mar 2021]. Available from: https://www.w3.org/WAI/WCAG21/quickref/?versions=2.0.

[43] Web Accessibility Initiative (WAI). Forms Concepts. In: Web Accessibility Tutorials: Guidance on how to create websites that meet WCAG [Internet]. 2019 [cited 11 Mar 2021]. Available from: https://www.w3.org/WAI/tutorials/forms/.

[44] WebAIM. Alternative Text. In: WebAIM: Web Accessibility in Mind [Internet]. 2019 [cited 11 Mar 2021]. Available from: https://webaim.org/techniques/alttext/.

[45] WebAIM. The WebAIM Million—An annual accessibility analysis of the top 1,000,000 home pages. In: WebAIM: Web Accessibility in Mind [Internet]. 2020 [cited 11 Mar 2021]. Available from: https://webaim.org/projects/million/.

[46] European Union. General Data Protection Regulation (GDPR). In: General Data Protection Regulation (GDPR) [Internet]. 2018 [cited 11 Mar 2021]. Available from: https://gdpr-info.eu/.

[47] McCoy O. A legislative comparison: US vs. EU on data privacy. In: European Interactive Digital Advertising Alliance (EDAA) [Internet]. 31 Mar 2020 [cited 11 Mar 2021]. Available from: https://edaa.eu/a-legislative-comparison-us-vs-eu-on-data-privacy/.

[48] Termly Legal Team. Privacy Policy Template. In: Termly [Internet]. 2020 [cited 11 Mar 2021]. Available from: https://termly.io/resources/templates/privacy-policy-template/.

[49] Michener WK. Ten Simple Rules for Creating a Good Data Management Plan. Bourne PE, editor. PLoS Comput Biol. 2015;11:e1004525. doi: 10.1371/journal.pcbi.1004525 26492633 PMC4619636

[50] Zipper SC, Stack Whitney K, Deines JM, Befus KM, Bhatia U, Albers SJ, et al. Balancing Open Science and Data Privacy in the Water Sciences. Water Resour Res. 2019;55:5202–11. doi: 10.1029/2019WR025080

[51] Carroll SR, Garba I, Figueroa-Rodríguez OL, Holbrook J, Lovett R, Materechera S, et al. The CARE Principles for Indigenous Data Governance. Data Sci J. 2020;19:43. doi: 10.5334/dsj-2020-043

[52] Meyer MN. Practical Tips for Ethical Data Sharing. Adv Methods Pract Psychol Sci. 2018;1:131–44. doi: 10.1177/2515245917747656

[53] Phillips M, Knoppers BM. Whose Commons? Data Protection as a Legal Limit of Open Science. J Law Med Ethics. 2019;47:106–11. doi: 10.1177/1073110519840489 30994061

[54] Society of Research Software Engineering. [cited 26 Aug 2021]. Available from: https://society-rse.org/.

[55] Research Software Groups. In: Research Software Engineers Association [Internet]. [cited 26 Aug 2021]. Available from: https://rse.ac.uk/community/research-software-groups-rsgs/.

[56] Research Software Engineers International. Introducing the International Council of RSE Associations. 27 Jan 2021 [cited 26 Aug 2021]. Available from: http://researchsoftware.org/2021/01/27/introducing-the-international-council-of-RSE-associations.html.

[57] Perez-Riverol Y, Gatto L, Wang R, Sachsenberg T, Uszkoreit J, Leprevost F da V, et al. Ten Simple Rules for Taking Advantage of Git and GitHub. Markel S, editor PLOS Computational Biology 2016;12:e1004947. doi: 10.1371/journal.pcbi.1004947 27415786 PMC4945047

[58] Lowndes JSS, Best BD, Scarborough C, Afflerbach JC, Frazier MR, O’Hara CC, et al. Our path to better science in less time using open data science tools. Nature Ecology & Evolution. 2017;1:0160. doi: 10.1038/s41559-017-0160 28812630

[59] Bryan J. Excuse Me, Do You Have a Moment to Talk About Version Control? Am Stat. 2018;72:20–7. doi: 10.1080/00031305.2017.1399928

[60] Foster C, Planella D, Gragnola J, Davis T. Issue and Kanban boards project management guidelines. In: GitLab [Internet]. 2020 [cited 12 Mar 2021]. Available from: https://about.gitlab.com/handbook/marketing/project-management-guidelines/boards/.

[61] Wizemann T, Ncha S, Gee AW, Shore C, editors. Enhancing Scientific Reproducibility in Biomedical Research Through Transparent Reporting: Proceedings of a Workshop. Washington, DC: The National Academies Press; 2020. doi: 10.17226/25627 32207888

[62] Chief Information Officers (CIO) Council. Open Data Policy—Managing Information as an Asset. In: Project Open Data [Internet]. 2019 [cited 12 Mar 2021]. Available from: https://github.com/project-open-data/project-open-data.github.io.

[63] American Geophysical Union (AGU). Data Policy. In: AGU Policy: Data [Internet]. 2016 [cited 12 Mar 2021]. Available from: https://www.agu.org/Publish-with-AGU/Publish/Author-Resources/Policies/Data-policy.

[64] Public Library of Science (PLOS) ONE. Data Availability. In: Public Library of Science (PLOS) ONE [Internet]. 2020 [cited 12 Mar 2021]. Available from: https://journals.plos.org/plosone/s/data-availability.

[65] Konigi. Schedule and Cost Summary Calculator. [cited 26 Aug 2021]. Available from: https://konigi.com/tools/schedule-and-cost-summary-calculator/.

[66] Everson M. Web Development Project Estimator 2021. Available from: https://github.com/astuteo/estimator.

[67] Ivan I, Despa ML. Estimating Maintenance Cost for Web Applications. IE. 2016;20:34–43. doi: 10.12948/issn14531305/20.4.2016.04

[68] Software Sustainability Institute. Writing and using a software management plan. 2021 [cited 13 Aug 2021]. Available from: https://www.software.ac.uk/resources/guides/software-management-plans.

[69] Anderson W, Morris E, Smith D, Ward MC. COTS and Reusable Software Management Planning: A Template for Life-Cycle Management: Fort Belvoir, VA: Defense Technical Information Center; 2007 Oct. doi: 10.21236/ADA473976

[70] Schultheiss SJ. Ten Simple Rules for Providing a Scientific Web Resource. PLoS Comput Biol. 2011;7:e1001126. doi: 10.1371/journal.pcbi.1001126 21637800 PMC3102757

[71] Sharan M, Brown A, Ball W, Krikler B. Nudging towards a better default for open source project ownership. 9 Jun 2021 [cited 13 Aug 2021]. Available from: https://www.software.ac.uk/blog/2021-06-09-nudging-towards-better-default-open-source-project-ownership.

[72] Lee BD. Ten simple rules for documenting scientific software. PLoS Comput Biol. 2018;14:e1006561. doi: 10.1371/journal.pcbi.1006561 30571677 PMC6301674

[73] Taschuk M, Wilson G. Ten simple rules for making research software more robust. PLoS Comput Biol. 2017;13:e1005412. doi: 10.1371/journal.pcbi.1005412 28407023 PMC5390961

[74] Parker MS, Burgess AE, Bourne PE. Ten simple rules for starting (and sustaining) an academic data science initiative. PLoS Comput Biol. 2021;17:e1008628. doi: 10.1371/journal.pcbi.1008628 33600414 PMC7891724

[75] Nüst D, Sochat V, Marwick B, Eglen SJ, Head T, Hirst T, et al. Ten simple rules for writing Dockerfiles for reproducible data science. Markel S, editor PLOS Computational Biology. 2020;16:e1008316. doi: 10.1371/journal.pcbi.1008316 33170857 PMC7654784

[76] Framework for Open and Reproducible Research Training (FORRT). Welcome to FORRT. In: Framework for Open and Reproducible Research Training (FORRT) [Internet]. 2021 [cited 12 Mar 2021]. Available from: https://forrt.org/.

[77] rOpenSci. A guide to enhancing reproducibility in scientific results and writing. In: Reproducibility in Science [Internet]. 2020 [cited 12 Mar 2021]. Available from: https://ropensci.github.io/reproducibility-guide/.

[78] ISO/IEC 14764:2006, Software Engineering, Software Life Cycle Processes, Maintenance. [cited 26 Aug 2021]. Available from: https://www.iso.org/obp/ui/#iso:std:iso-iec:14764:ed-2:v1:en.

[79] Ivan I, Luca N-C, Despa ML, Budacu E. Maintenance-Ready Web Application Development IE. 2016;20:28–36. doi: 10.12948/issn14531305/20.3.2016.03

[80] Rahman HU, Raza M, Afsar P, Khan M, Iqbal N, Khan HU. Making the Sourcing Decision of Software Maintenance and Information Technology. IEEE Access. 2021;9:11492–510. doi: 10.1109/ACCESS.2021.3051023

[81] Best Practices for Maintainers. In: Open Source Guides [Internet]. 2021 [cited 26 Aug 2021]. Available from: https://opensource.guide/best-practices/.

[82] Russell A, Vinsel L. Hail the maintainers. In: Aeon Essays [Internet]. 7 Apr 2016 [cited 26 Aug 2021]. Available from: https://aeon.co/essays/innovation-is-overvalued-maintenance-often-matters-more.

[83] Prlić A, Procter JB. Ten Simple Rules for the Open Development of Scientific Software. PLoS Comput Biol. 2012;8:e1002802. doi: 10.1371/journal.pcbi.1002802 23236269 PMC3516539

[84] Sandve GK, Nekrutenko A, Taylor J, Hovig E. Ten Simple Rules for Reproducible Computational Research. PLoS Comput Biol. 2013;9:e1003285. doi: 10.1371/journal.pcbi.1003285 24204232 PMC3812051

[85] List M, Ebert P, Albrecht F. Ten Simple Rules for Developing Usable Software in Computational Biology. PLoS Comput Biol. 2017;13:e1005265. doi: 10.1371/journal.pcbi.1005265 28056032 PMC5215831

[86] Helmy M, Crits-Christoph A, Bader GD. Ten Simple Rules for Developing Public Biological Databases. PLoS Comput Biol. 2016;12:e1005128. doi: 10.1371/journal.pcbi.1005128 27832061 PMC5104318

[87] Bourne PE, Polka JK, Vale RD, Kiley R. Ten simple rules to consider regarding preprint submission. PLoS Comput Biol. 2017;13:e1005473. doi: 10.1371/journal.pcbi.1005473 28472041 PMC5417409

[88] Mozilla. Best Practices Working Open. In: Open Leadership Training Series [Internet]. 2020 [cited 12 Mar 2021]. Available from: https://mozilla.github.io/open-leadership-training-series/.

[89] Lowndes JSS. Audience building and strategic planning. In: Openscapes [Internet]. 2021 [cited 12 Mar 2021]. Available from: https://www.openscapes.org/blog/2021/01/06/audience-building/.

[90] Smith AM, Katz DS, Niemeyer KE. Software citation principles PeerJ Comput Sci. 2016;2:e86. doi: 10.7717/peerj-cs.86

[91] Carlson D, Oda T. Editorial: Data publication–*ESSD* goals, practices and recommendations. Earth System Science Data. 2018;10:2275–8. doi: 10.5194/essd-10-2275-2018

