# Supplementary figures and images for: Ten simple rules for researchers who want to develop web apps
Source: PLoS Comput Biol. 2022 Jan 6;18(1):e1009663. doi: 10.1371/journal.pcbi.1009663 (PMC8735566; doi:10.1371/journal.pcbi.1009663)

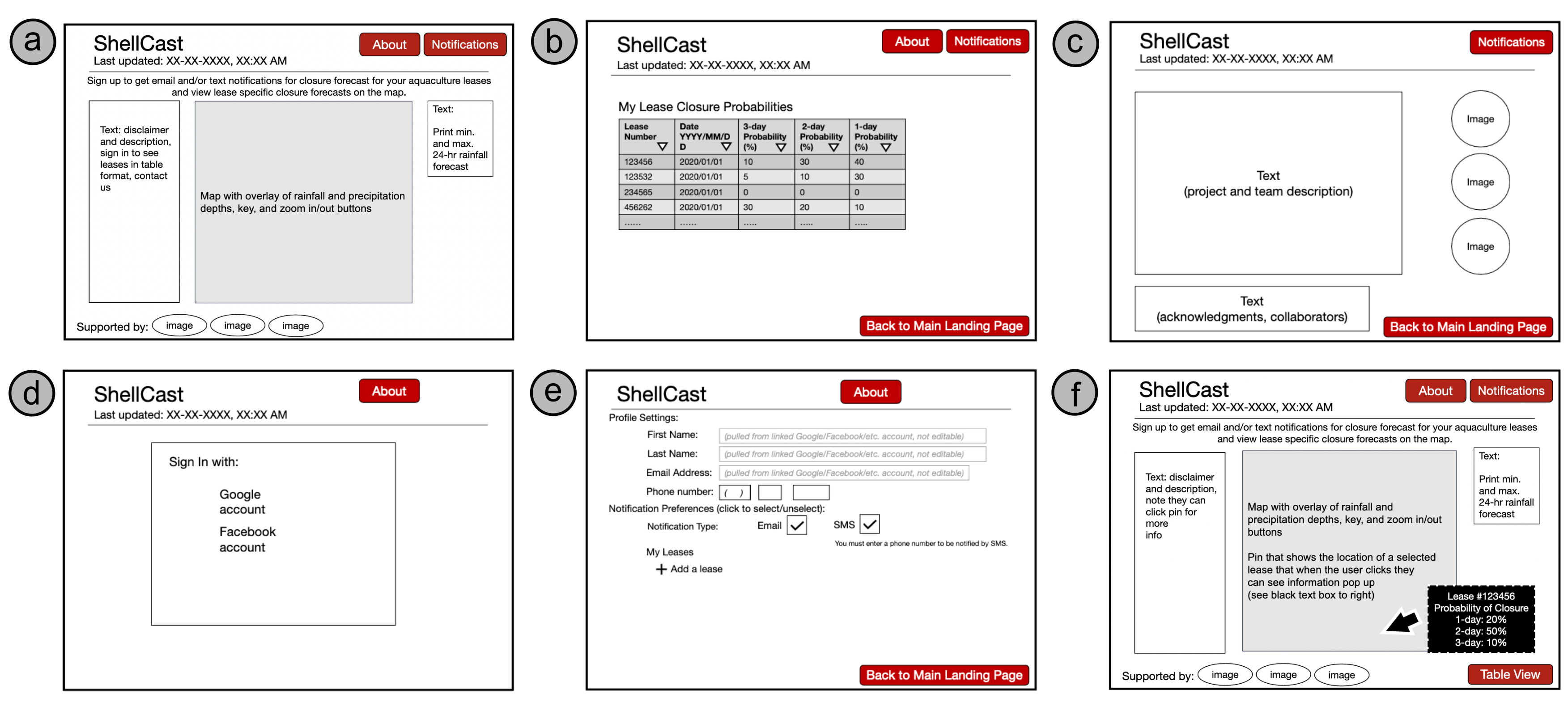

Supplement: S1 Fig — ShellCast wireframes, including (a) main page map view when user is not signed in, (b) main page table view, (c) ShellCast “About” page, (d) user login page, (e) user notifications/profile page, and (f) main page map view when user is signed in (can see lease pin and click pin to see lease-specific information). (PNG) [file pcbi.1009663.s001.png]
