# Supplementary material for: Ten simple rules for researchers who want to develop web apps
Source: PLoS Comput Biol. 2022 Jan 6;18(1):e1009663. doi: 10.1371/journal.pcbi.1009663 (PMC8735566; doi:10.1371/journal.pcbi.1009663)
Supplement: S1 Text — RFP, request for proposals. (DOCX) [file pcbi.1009663.s002.docx]

**S1 Text.** First version of our request for proposals (RFP) contract “Scope of Work” section.

1. Scope of Work

The backend of the web application shall be created with Python (e.g. Django). The Contractor shall propose a framework or approach for the front-end of the application, as well as the SMS alert service. The proposal shall detail how testing is integrated in the development process, as well as how blocking bugs are identified and addressed early.

1.1 Features of the web application backend

- Pulls the NDFD Probability of Precipitation (PoP) and Quantitative Precipitation Forecast (QPF) products twice daily using the National Weather Service API; in the event that the National Weather Service API is down, the application shall post a notification on the web application interface.
- Calculates the probability of closure for each growing area in North Carolina (equations and values to be provided by the University).
- If the probability of closure exceeds a certain threshold, the application sends a SMS message and/or email to users based on their subscription preferences.
- Usage tracking (e.g. number of SMS messages sent, number of subscribers).

1.2 Features of the web application frontend (webpage)

- Description and history of web application.
- User registration: users submit contact information, shellfish growing areas, and notification preferences.
- User opt-out: users can unsubscribe from notifications.
- Map showing all shellfish growing areas in NC as well as closure probabilities. The university will supply the underlying files to create the map. The awarded contractor will create the map displayed on the application’s frontend.

1.3 Open source requirements

The Python code used to create the web application must be open source. The web application will have either a GNU General Public License or Creative Commons Attribution 3.0 Unported (“”CC-BY”) License. The Contractor must comply with all U.S. Federal Open Data policies (see https://project-open-data.cio.gov/ for more details).

1.4 Timeline

Project starts: April 1, 2020

Fully functional application is developed and summary report due: June 30, 2020

Users test application: August 1, 2020 – September 30, 2021

Contractor receives requests for changes from the University: October 15, 2021

Contractor finalizes web application and submits final documentation: January 15, 2022

1.5 Progress Reporting

Awarded contractor will provide monthly progress reports via e-mail and video conferencing with the university team responsible for this project. The university will set up the conference calls monthly. Note any research data associated with this project must remain within the contiguous United States of America.
