# Supplementary material for: Ten simple rules for researchers who want to develop web apps
Source: PLoS Comput Biol. 2022 Jan 6;18(1):e1009663. doi: 10.1371/journal.pcbi.1009663 (PMC8735566; doi:10.1371/journal.pcbi.1009663)
Supplement: S2 Text — RFP, request for proposals. (DOCX) [file pcbi.1009663.s003.docx]

**S2 Text.** Final version of our request for proposals (RFP) contract “Scope of Work” section.

1. Scope of Work

The backend of the web application shall be created using a Linux, Apache, MySQL, PHP environment or an alternative open source framework that is compatible with goals outlined in Section 1.1 as well as the University's web hosting service. The Contractor shall propose an open-source framework for the frontend of the web application that is consistent with the goals outlined in Section 1.2. All code shall be committed to a GitHub repository created by the University. The Contractor shall use MailChimp as the SMS and email gateway. The University shall host and backup the web application (specifications outlined at https://oit.ncsu.edu/campus-it/web-services/). Costs associated with hosting and SMS/email alert notifications will be directly managed by the University and should not be included in the proposal budget. The proposal shall detail how testing is integrated in the development process, as well as how blocking bugs are identified and addressed early. The Contractor is expected to address web application bugs and updates from the project start date until January 15, 2022; the proposal should include an estimated cost to the University for any maintenance required over the duration of the project.

Draft wireframes of front-end pages are provided below. These wireframes are intended to communicate overall page structure, but the appearance shall adhere to the policies and standards outlined in Section 1.2 below. The awarded Contractor is encouraged to suggest aesthetic improvements to the overall design.

*(We inserted wireframes here: see Figure S1.)*

1.1 Features of the web application backend

- The University will write R and Python scripts to pull Probability of Precipitation (PoP) and Quantitative Precipitation Forecast (QPF) products from the National Weather Service (NWS) API (https://graphical.weather.gov/xml/rest.php), do calculations, and export results to the shellfish growing area closure probabilities table (see below). The tables will be used to update the web application frontend.
- R and Python scripts shall be scheduled to run every 12 hours for each shellfish growing area in North Carolina. If the NWS API is down, the web application shall display the most recent closure probabilities information along with the status of the NWS API server.
- If the maximum probability of closure in a 3-day window exceeds a certain threshold, the application shall send a plain text SMS and/or email alert notification to users based on their subscription preferences. When users subscribe to SMS notifications for multiple shellfish growing areas that are under alert, they shall receive one notification per shellfish growing area. For users who subscribe to email alert notifications for multiple shellfish growing areas that are under alert, they shall receive one consolidated email notification that includes all shellfish growing areas they subscribe to. SMS messages are expected to be approximately 50-75 characters long, e.g. “70% of closure in SGA A1 within next 3 days”. SMS and email messages are expected to be sent in a timely manner.
- The web application database shall consist of the following tables and columns at a minimum:
  - Table: Subscriber contact information. Columns: Unique subscriber ID number, first name, last name, phone number, email address, date-time subscribed for SMS, date-time subscribed for emails, date-time unsubscribed to SMS, date-time unsubscribed to emails.
  - Table: User subscription preferences. Columns: Unique subscriber ID number, user first and last name, shellfish growing area, SMS (yes/no), email (yes/no). Records will repeat for each shellfish growing area a user subscribes to.
  - Table: Shellfish growing area closure probabilities. Columns: shellfish growing area, date-time, 3-day probability, 2-day probability, 1-day probability, color classification maximum probability in 3-day window, color classification for 3-day probability, color classification for 2-day probability, color classification for 1-day probability. This table shall log all calculated probabilities in individual rows; existing rows shall not be overwritten.
  - Table: Alert logging. Columns: Unique subscriber ID, date-time, alert type, shellfish growing area, third party SMS/email gateway confirmation, alert message plain text.
  - Table: Shellfish growing areas. Columns: Shellfish growing area, shellfish growing area status (approved, conditionally approved, prohibited), rainfall threshold, shellfish growing area shapefile path. Information populated in this table will be provided by the University and shall be readily editable by the University.
  - Table: Disclaimers. Columns: Disclaimer text, corresponding page on frontend. Disclaimer text will be provided by the University and shall be readily editable by the University.

1.2 Features of the web application frontend (web application)

The frontend shall be created with a technology that operates in a Linux Apache MySQL PHP environment.

- Main Landing Page: Includes header with the web application name and logo, which will be provided by the University; buttons for “Sign Up”, “Sign In”, and “About” pages; disclaimer text, which is pulled from the disclaimer table in the database; text showing the date and time when the probabilities and frontend were last updated; text explaining that those interested in receiving alert notifications should sign up. Below the header, a map overlaid on a widget or basemap occupies the majority of the page. The map shows all shellfish growing areas in North Carolina, and the shellfish growing area polygons are colored based on up-to-date closure probabilities. If a user selects a growing area, the name and closure probabilities (1-day, 2-day, 3-day) are shown. Users can toggle between 3-day, 2-day, and 1-day closure probability maps, with the max of the 1-, 2-, and 3-day probabilities set as the default (described as “closure probability within next 3 days”). Users can zoom in and out of the map. The University will supply all shellfish growing area spatial boundaries in one shapefile (.shp), as well as the metadata needed to create the map overlay. Closure probabilities shall be calculated on the backend and updated twice daily. Below the map, images and text are included to acknowledge organizations affiliated with the web application; the text and images will be provided by the University and shall be editable. There is also a “Table View” button at the bottom of the page to view the Main Landing Page data as a sortable table.
- About: Description and history of the web application. Includes text and images, all of which will be provided by the University. The text and images shall be editable by the University. A “Back to Main Landing Page” button at the bottom of this page will return the user to the non-table view of the Main Landing Page.
- Sign Up: Users register using third-party social media accounts (Google and Facebook are required at a minimum). The Sign Up page pulls disclaimer text from the disclaimer table in the database. More details described in 3.2.5 below.
- Sign In: Users log in using a third-party social media account (Google and Facebook are required at a minimum) and can view their current account profile. The Sign In page pulls disclaimer text from the disclaimer table in the database. More details described in 3.2.5 below.
- User subscription preferences after Sign Up and Sign In: Once they sign up or sign in, users can view and/or make changes to their preferences. First name, last name, and email address are pulled from the third-party social media account and are not editable by the user. Phone number, alert notification preference (i.e., SMS and/or email), and shellfish growing areas shall be editable by the user. Users must enter a phone number to check that they want to receive SMS alert notifications and can choose to subscribe to multiple shellfish growing areas. A “Back to Main Landing Page” button at the bottom of this page will return the user to the non-table view of the Main Landing Page. The user subscription preferences page pulls disclaimer text from the disclaimer table in the database.
- Main Landing Page in Table View: Information presented on the main landing page (shellfish growing areas, probabilities of closure) shall be in a sortable table for easy viewing with screen readers and those who want to view the data in this format. Data to populate the table will come from the shellfish growing area closure probabilities table. A “Back to Main Landing Page” button at the bottom of this page will return the user to the non-table view of the Main Landing Page.
- Additional features: The web application shall meet the Revised Section 508 standards (https://www.access-board.gov/guidelines-and-standards/communications-and-it/about-the-ict-refresh/final-rule/text-of-the-standards-and-guidelines) incorporating the Web Content Accessibility Guidelines (WCAG) 2.0 A and AA. If possible, a current Voluntary Product Accessibility Template (VPAT) 2.0 or later detailing how this product will meet the aforementioned accessibility standards shall be provided. The web application shall meet the University’s branding requirements (https://brand.ncsu.edu/). The web application shall utilize responsive design and be readily viewable on a mobile device. Each page of the web application shall incorporate Firebase analytics.

1.3 Open source requirements

All code used to create the web application shall be open source. The web application shall have either a GNU General Public License or Creative Commons Attribution 3.0 Unported (“”CC-BY”) License. The Contractor shall comply with all U.S. Federal Open Data policies (see https://project-open-data.cio.gov/ for more details).

1.4 Timeline

Project starts: April 1, 2020

Fully functional application is developed and summary report due: June 30, 2020

Users test application: August 1, 2020 – September 30, 2021

Contractor receives requests for changes from the University: October 15, 2021

Contractor finalizes web application and submits final documentation: January 15, 2022

1.5 Progress Reporting

Awarded Contractor will provide monthly progress reports via e-mail and video conferencing with the University team responsible for this project. The University will set up the conference calls monthly.

1. 6 Data and web application ownership

Note any research data associated with this project shall remain within the contiguous United States of America. *Ownership of all code, design, and databases shall be relinquished to the University.*
