# Supplementary material for: Ten simple rules for researchers who want to develop web apps
Source: PLoS Comput Biol. 2022 Jan 6;18(1):e1009663. doi: 10.1371/journal.pcbi.1009663 (PMC8735566; doi:10.1371/journal.pcbi.1009663)
Supplement: S3 Text — (DOCX) [file pcbi.1009663.s004.docx]

**S3 Text.** “Phase 0” ShellCast user testing survey questions. Pre-test questions were administered before testing started and post-test questions were administered a week later, when web app testing was finished.

*Pre-Test Questions*

1. What browser did you use to view ShellCast? (multiple choice: Firefox, Safari, Chrome, Internet Explorer, other - fill in)
2. Where did you view ShellCast? (multiple choice: phone, tablet, computer, other - fill in)
3. To the best of your ability, please describe the purpose of ShellCast from memory. We ask that you write this response without returning to the About page because we’re interested in learning which parts of the description stood out to you. (long answer)
4. To the best of your ability, please describe the information shown on the main landing page map from memory. We ask that you write this response without returning to the main landing page as we’re interested in learning if the content on the map was memorable. (long answer)
5. At any point while reviewing the main landing page did you feel confused? (multiple choice: yes, no)
6. If you answered yes to question #5, please describe where and why you were confused? If you have to look back at ShellCast, please do. (long answer)
7. Did you interact with the map by clicking on growing areas? (multiple choice: yes, no, I’m not sure)
8. Did you interact with the map by clicking on 1-day, 2-day, and/or 3-day forecast options? (multiple choice: yes, no, I’m not sure)
9. At any point while reviewing the About page did you feel confused? (multiple choice: yes, no)
10. If you answered yes to question #9, please describe where and why you were confused? If you have to look back at ShellCast, please do. (long answer)
11. Did you encounter any difficulties when creating an account? (multiple choice: yes, no)
12. If you answered yes to question #11, please explain. (long answer)
13. Did you encounter any difficulties when adding leases? (multiple choice: yes, no)
14. If you answered yes to question #13, please explain. (long answer)
15. Overall, what is your opinion of the ShellCast web application? (Was the user interface simple, straightforward, and easy to use or complex, full of jargon and hard to understand?) (long answer)
16. Please suggest two or more ways we can improve the ShellCast web application. (long answer)
17. Please add any other comments or questions about the ShellCast web application. (Including typos, confusing words/wording, something isn't working properly, and additional functionality is needed.) (long answer)

*Post-Test Questions*

1. Did you choose to receive text message notifications for any of your leases? (multiple choice: yes, no)
2. If you answered yes to question #1, how many text message notifications did you receive over the course of the week? (short answer)
3. If you answered yes to question #1, what is your mobile phone service provider? (multiple choice: AT&T, Verizon, Sprint, other - fill in)
4. Did you choose to receive email notifications for any of your leases? (multiple choice: yes, no)
5. If you answered yes to question #4, how many email notifications did you receive over the course of the week? (short answer)
6. Did you use the link provided in the notification to return to the ShellCast web application? (multiple choice: yes, no, I’m not sure)
7. Describe two things you did not like about ShellCast. (long answer)
8. Describe two things you liked about ShellCast. (long answer)
9. Describe two things you are still confused about after using ShellCast. (long answer)
10. The term “1-day” was used in several places on the main page of ShellCast as well as in ShellCast notifications. What did the “1-day” forecast mean to you? (multiple choice: 1-day meant today, 1-day meant tomorrow, I’m not sure what 1-day meant, other - fill in)
11. The term “3-day” was used in several places on the main page of ShellCast as well as in ShellCast notifications. What did the “3-day” forecast mean to you? (multiple choice: 3-day meant a three-day period (cumulative), 3-day meant one day 3 days from now, I’m not sure what 3-day meant, other - fill in)
12. Overall, what is your opinion of the ShellCast notifications? (Were the notifications simple, straightforward, and easy to follow or complex, full of jargon and hard to understand?) (long answer)
13. Please suggest two or more ways we can improve ShellCast notifications. (long answer)
14. Please add any other comments or questions about the ShellCast notifications. (Including typos, confusing words/wording, something isn't working properly, and additional functionality is needed.) (long answer)
