# Supplementary material for: Ten simple rules for researchers who want to develop web apps
Source: PLoS Comput Biol. 2022 Jan 6;18(1):e1009663. doi: 10.1371/journal.pcbi.1009663 (PMC8735566; doi:10.1371/journal.pcbi.1009663)
Supplement: S5 Text — (DOCX) [file pcbi.1009663.s006.docx]

**S5 Text.** ShellCast privacy policy.

# Introduction

Last updated December 5, 2020

ShellCast (“we” or “us” or “our”) respects the privacy of our users (“user” or “you”). This Privacy Policy explains how we collect, use, disclose, and safeguard your information when you visit our website https://go.ncsu.edu/shellcast, including any other media form, media channel, mobile website, or mobile application related or connected thereto (collectively, the “Site”). Please read this privacy policy carefully. If you do not agree with the terms of this privacy policy, please do not access the site.

We reserve the right to make changes to this Privacy Policy at any time and for any reason. We will alert you about any changes by updating the “Last Updated” date of this Privacy Policy. Any changes or modifications will be effective immediately upon posting the updated Privacy Policy on the Site, and you waive the right to receive specific notice of each such change or modification.

You are encouraged to periodically review this Privacy Policy to stay informed of updates. You will be deemed to have been made aware of, will be subject to, and will be deemed to have accepted the changes in any revised Privacy Policy by your continued use of the Site after the date such revised Privacy Policy is posted.

# Collection of Your Information

We may collect information about you in a variety of ways. The information we may collect on the Site includes:

## Personal Data

Personally identifiable information, such as your email address, telephone number, and mobile phone service provider, that you voluntarily give to us when you register with the Site. You are under no obligation to provide us with personal information of any kind, however your refusal to do so may prevent you from using certain features of the Site.

## Google Permissions

If you choose to register with the Site through Google, then we will record the email address associated with your Google account.

## Facebook Permissions

If you choose to register with the Site through Facebook, then we will record the email address associated with your Facebook account.

# Use of Your Information

Having accurate information about you permits us to provide you with a smooth, efficient, and customized experience. Specifically, we may use information collected about you via the Site to:

- Assist law enforcement and respond to subpoena.
- Compile anonymous, aggregate statistical data and analysis for use internally or publicly.
- Create and manage your account.
- Email you regarding your account.
- Monitor and analyze usage and trends to improve your experience with the Site.
- Notify you of updates to the Site.
- Request feedback and contact you about your use of the Site.
- Send you notifications.

# Disclosure of Your Information

We may share information we have collected about you in certain situations. Your information may be disclosed as follows:

## By Law or to Protect Rights

If we believe the release of information about you is necessary to respond to legal process, to investigate or remedy potential violations of our policies, or to protect the rights, property, and safety of others, we may share your information as permitted or required by any applicable law, rule, or regulation. This includes exchanging information with other entities for fraud protection and credit risk reduction.

## Third-Party Service Providers

We may share your information with third parties that perform services for us or on our behalf, including email delivery and hosting services.

# Third-Party Websites

The Site may contain links to third-party websites and applications of interest that are not affiliated with us. Once you have used these links to leave the Site, any information you provide to these third parties is not covered by this Privacy Policy, and we cannot guarantee the safety and privacy of your information. Before visiting and providing any information to any third-party websites, you should inform yourself of the privacy policies and practices (if any) of the third party responsible for that website, and should take those steps necessary to, in your discretion, protect the privacy of your information. We are not responsible for the content or privacy and security practices and policies of any third parties, including other sites, services or applications that may be linked to or from the Site.

# Security of Your Information

We use administrative, technical, and physical security measures to help protect your personal information. While we have taken reasonable steps to secure the personal information you provide to us, please be aware that despite our efforts, no security measures are perfect or impenetrable, and no method of data transmission can be guaranteed against any interception or other type of misuse. Any information disclosed online is vulnerable to interception and misuse by unauthorized parties. Therefore, we cannot guarantee complete security if you provide personal information.

# Policy For Children

We do not knowingly solicit information from or market to children under the age of 13. If you become aware of any data we have collected from children under age 13, please contact us using the contact information provided at the end of this document.

# Controls For Do-Not-Track Features

Most web browsers and some mobile operating systems include a Do-Not-Track (“DNT”) feature or setting you can activate to signal your privacy preference not to have data about your online browsing activities monitored and collected. No uniform technology standard for recognizing and implementing DNT signals has been finalized. As such, we do not currently respond to DNT browser signals or any other mechanism that automatically communicates your choice not to be tracked online. If a standard for online tracking is adopted that we must follow in the future, we will inform you about that practice in a revised version of this Privacy Policy. Most web browsers and some mobile operating systems include a Do-Not-Track (“DNT”) feature or setting you can activate to signal your privacy preference not to have data about your online browsing activities monitored and collected. If you set the DNT signal on your browser, we will respond to such DNT browser signals.

# Options Regarding Your Information

## Account Information

You may at any time review or change the information in your account or terminate your account by:

- Logging into your account settings and updating your account.
- Contacting us using the contact information provided at the end of this document.

Upon your request to terminate your account, we will deactivate or delete your account and information from our active databases. However, some information may be retained in our files to prevent fraud, troubleshoot problems, assist with any investigations, enforce our Terms of Use and/or comply with legal requirements.

## Emails and Communications

If you no longer wish to receive correspondence, emails, or other communications from us, you may opt-out by:

- Noting your preferences at the time you register your account with the Site.
- Logging into your account settings and updating your preferences.
- Contacting us using the contact information provided at the end of this document.

If you no longer wish to receive correspondence, emails, or other communications from third parties, you are responsible for contacting the third party directly.

# California Privacy Rights

California Civil Code Section 1798.83, also known as the “Shine The Light” law, permits our users who are California residents to request and obtain from us, once a year and free of charge, information about categories of personal information (if any) we disclosed to third parties for direct marketing purposes and the names and addresses of all third parties with which we shared personal information in the immediately preceding calendar year. If you are a California resident and would like to make such a request, please submit your request in writing to us using the contact information provided at the end of this document.

If you are under 18 years of age, reside in California, and have a registered account with the Site, you have the right to request removal of unwanted data that you publicly post on the Site. To request removal of such data, please contact us using the contact information provided at the end of this document, and include the email address associated with your account and a statement that you reside in California. We will make sure the data is not publicly displayed on the Site, but please be aware that the data may not be completely or comprehensively removed from our systems.

# Contact Us

If you have questions or comments about this Privacy Policy, please contact us at: shellcastapp@ncsu.edu
